# Predictors of 30-Day Mortality in Trauma: A Single-Center Retrospective Exploratory Study

**DOI:** 10.3390/life15121929

**Published:** 2025-12-17

**Authors:** Irina-Anca Eremia, Cătălin-Alexandru Anghel, Horia Alexandru Nica, Eduard-Alexandru Eremia, Ionuț-Lucian Antone-Iordache, Adrian-Gabriel Borcan, Daniel Iulian Voiculescu, Silvia Nica

**Affiliations:** 1 Department of Family Medicine III, Carol Davila University of Medicine and Pharmacy, 050474 Bucharest, Romania; irina.eremia@umfcd.ro; 2 Emergency Department, Emergency University Hospital Bucharest, 050098 Bucharest, Romania; 3 Doctoral School, Carol Davila University of Medicine and Pharmacy, 050474 Bucharest, Romania; ionut-lucian.antone-iordache@drd.umfcd.ro; 4 Faculty of Medicine, Carol Davila University of Medicine and Pharmacy, 050474 Bucharest, Romania; horia-alexandru.nica2023@stud.umfcd.ro (H.A.N.); adrian-gabriel.borcan0720@stud.umfcd.ro (A.-G.B.); 5 Faculty of Medicine, Ludwig Maximilian University of Munich, 80336 Munich, Germany; ere.edu01@gmail.com; 6 Radiotherapy Department, Colțea Clinical Hospital, 030167 Bucharest, Romania; 7 Department of General Surgery, Faculty of Medicine, Carol Davila University of Medicine and Pharmacy, 020021 Bucharest, Romania; daniel.voiculescu@umfcd.ro; 8 Department of Emergency and First Aid, Carol Davila University of Medicine and Pharmacy, Emergency University Hospital Bucharest, 050474 Bucharest, Romania; silvia.nica@umfcd.ro

**Keywords:** trauma patients, trauma scores, 30-day mortality, mortality predictors, exploratory prognostic model

## Abstract

*Background and Objectives*: Trauma remains a leading global cause of preventable mortality, and outcome prediction tools are essential for both triage and resource allocation. Timely and effective medical response to trauma patients is of the essence. While there are already some widely accepted trauma scores, our aim was to build a more precise model tailored to our cohort as a framework for future research. *Materials and Methods*: A retrospective cohort of 91 patients was analyzed, using several clinical and paraclinical factors to build a logistic regression model that predicted 30-day mortality. *Results*: After adjusting for collinearity, our final multiple regression model, comprising systolic blood pressure, glycemia, urea serum levels and number of fractures, showed an excellent model fit (McFadden R^2^ = 0.682; AUC = 0.94) for predicting 30-day mortality. Systolic blood pressure was significantly associated with mortality (OR = 0.944, 95% CI: 0.920–0.969). In our cohort it seems to surpass the performance of The Trauma and Injury Severity Score (McFadden R^2^ = 0.56; AUC = 0.90). *Conclusions*: Commonly available clinical parameters could contribute to risk stratification, highlighting the prognostic importance of hemodynamic instability and metabolic response. This study was conducted on a small cohort as an exploratory analysis. Further research and validation based on multicentric cohorts are needed.

## 1. Introduction

Trauma represents a major global public health issue, accounting for approximately 4.4 million deaths, about 8% of all mortality. According to the World Health Organization (WHO), trauma is the leading cause of death among people under 35 years old, and the sixth across all age groups. The majority of fatalities are the consequence of road traffic collisions, followed by suicides, homicides and falls, either from the same level or from a height. The impact of these events is augmented by their unexpected and often preventable nature, as well as by the social and economic costs caused by premature loss of socially and professionally active individuals [1,2].

In Romania, the burden of severe trauma remains considerable, with mortality rates surpassing the European Union average across most mechanisms of injury. Eurostat data from 2018 to 2020 indicate a high road traffic-related mortality, including in the pediatric and adolescent population (0–17 years), while mortality from both intentional violence and suicide also exceeds the European average. These findings call for stronger prevention measures and for timely and effective medical response, especially in Level I Trauma Centers where critically injured patients are most often treated [1,3].

In the context of these alarming figures, the management of severely injured patients requires a rapid and objective assessment of injury severity from the moment of presentation to the Emergency Department (ED). Over the last few decades, several scoring systems have been developed, aimed at quantifying injury severity and estimating both vital and functional prognosis.

The Injury Severity Score (ISS) is an anatomical score first described in 1974 by Baker et al., derived from the Abbreviated Injury Scale (AIS). By assigning an AIS score (1–6) to the most severe injury in each anatomical region (head and neck, face, chest, abdomen, extremities and external) and summing the squares of the three highest scores from distinct regions, the ISS ranges between 0 and 75. Nowadays, it is one of the most frequently used indicators of trauma severity, applied in triage and prognosis evaluation, as well as for benchmarking trauma centers’ performance [4,5,6,7,8].

The Revised Trauma Score (RTS) was proposed in 1989 by Champion et al. as a simplified version of the original Trauma Score in order to improve the initial assessment of trauma patients. It uses three easy-to-measure clinical parameters: Glasgow Coma Scale (GCS), Systolic Blood Pressure (SBP) and Respiratory Rate (RR), each graded on a scale from 0 to 4. In practice, there are two calculation methods: in the prehospital setting, the sum of the coded values is used as a rapid mean of identifying critically injured patients, while in the hospital setting a weighted version is used, in which each parameter’s score is multiplied by statistically derived coefficients to yield a result capable of estimating survival probability [6,7,9,10,11].

The Trauma and Injury Severity Score (TRISS) was developed in 1987 as a predictive model of survival among trauma patients. This integrates anatomical parameters (ISS), clinical parameters (RTS) and the age and the mechanism of injury in a logistic regression equation and has become the gold standard for evaluating the quality of care in trauma centers and for comparing outcomes between hospitals or trauma systems against national reference standards [6,7,12,13,14].

Beyond established scores, which allow assessment of injury severity and prediction of mortality, specific tools have been developed in order to enable an early identification of patients at risk of life-threatening hemorrhage and to support rapid resuscitation management. Among these, the most widely used are the Assessment of Blood Consumption (ABC) score and the Trauma Associated Severe Hemorrhage (TASH) score. The ABC score relies on four simple clinical criteria that are immediately available at patient admission: penetrating mechanism of trauma, SBP ≤ 90 mmHg, Heart Rate (HR) ≥ 120 beats per minute, and positive Focused Assessment with Sonography for Trauma (FAST) examination [15,16,17,18]. In contrast, the TASH score integrates clinical, laboratory, and imaging parameters (sex, hemoglobin, base excess, SBP, HR, positive FAST for intra-abdominal fluid, clinically unstable pelvic fracture, open or dislocated femur fracture) into a more complex model with high predictive accuracy [18,19,20]. In addition to widely established international scores for predicting mortality and transfusion needs, there remains a necessity to evaluate models that more accurately reflect the epidemiological realities and clinical characteristics of different geographical regions. In this context, we considered it appropriate to explore the relevance of clinical factors available at the presentation and to assess the feasibility of developing a simplified, locally tailored prognostic model that would enable efficient triage, optimal resource allocation, and appropriate management during the critical early hours following trauma.

Despite the widespread international use of these prognostic trauma scores, data on their performance and applicability in the Romanian trauma population remain limited, as epidemiological particularities and emergency system organization may influence prognostic accuracy. In addition, given that, at least in the initial stage, cases are managed territorially, including in smaller centers, there is a clear need to develop simplified prognostic models based on clinical parameters readily available during initial assessment. This gap provided the rationale for the present study.

The aim of this study was to identify clinical factors associated with 30-day mortality in trauma patients and to compare the performance of established scores (ISS, RTS, TRISS, ABC score, TASH score) with that of a simplified logistic model derived from the analyzed cohort.

## 2. Materials and Methods

A retrospective, observational, non-interventional study was conducted in the Emergency Department of the University Emergency Hospital Bucharest. The study was conducted in accordance with the Declaration of Helsinki and approved by the Ethics Committee of the University Emergency Hospital, Bucharest, Romania (no. 71992/6/30 September 2025). Written informed consent for the anonymized use of clinical data for research purposes was obtained from all patients at hospital admission. Data were collected retrospectively from clinical evaluation sheets and from the hospital electronic information system.

All adults (≥18 years) presenting to the Emergency Department with trauma over a six-month period (between 1 January 2025 and 30 June 2025), with an ISS ≥ 8 and relevant clinical and paraclinical data available, were included in the analysis. We chose the ISS threshold of ≥8 to exclude minor trauma and focus on patients with clinically significant injuries. Although polytrauma is conventionally defined as ISS ≥16, we intentionally used a lower threshold to capture patients that, although not strictly meeting the anatomical criteria for polytrauma, may still experience unfavorable outcomes in real-world clinical practice.

Exclusion criteria consisted of readmissions for trauma, delayed presentations (≥24 h after the traumatic event), patients with non-survivable injuries at arrival, and cases with missing essential data. “Non-survivable injuries at arrival” referred to patients presenting with injuries incompatible with life, such as refractory cardiac arrest without return of spontaneous circulation or catastrophic lesions precluding meaningful stabilization. These patients were excluded because a complete assessment of injury severity and laboratory parameters was not feasible, preventing uniform data analysis.

**Figure 1:**
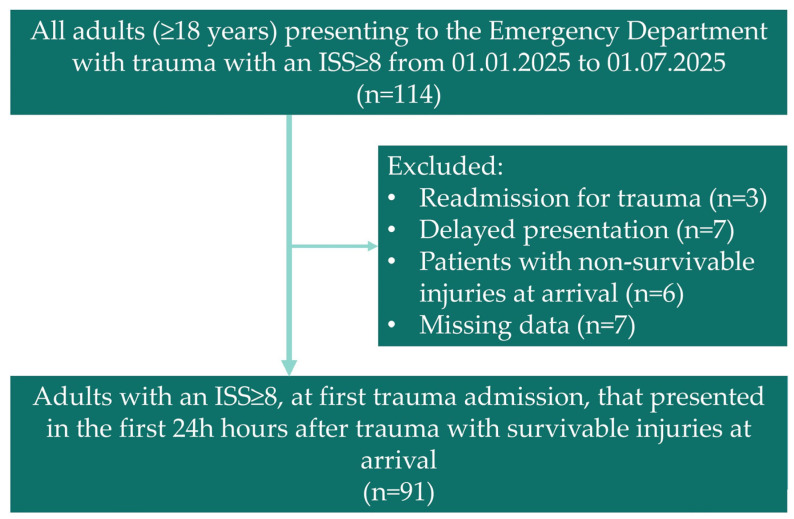
Flowchart showing the patients included in our study.

After applying these criteria, the final cohort comprised 91 patients out of the 114 considered (Figure 1).

For each patient, the following data were collected and analyzed: demographic characteristics (age, sex, comorbidities, mechanism of injury), initial clinical parameters (SBP, HR, RR, SpO2, GCS, ISS, RTS, TRISS, ABC score, TASH score), laboratory investigations (hemoglobin, platelet count, lactate, base deficit, blood glucose, urea, serum creatinine, International normalized ratio (INR) test, fibrinogen, Activated Partial Thromboplastin Time (aPTT)), imaging examinations performed, therapeutic interventions required, 30-day mortality, total length of hospital stay, and Intensive Care Unit (ICU) stay.

All clinical and laboratory parameters were recorded at Emergency Department admission during the initial patient assessment.

In this study, 30-day mortality was defined as death from any cause occurring within 30 days of the traumatic event, regardless of the site of injury.

For statistical analyses JASP Version 0.19.3 was used.

Categorical variables were presented as number of patients and frequencies (percentages), while continuous variables were presented as mean and standard deviation, when appropriate, also as median with interquartile range (IQR).

Cases with missing essential data required for score calculation or regression analysis were excluded using a complete-case approach, without data imputation. This approach may introduce a potential selection bias by excluding patients with incomplete data; however, the number of excluded cases was small, and the exclusion criteria were applied uniformly within the cohort.

Sample size for univariate logistic regressions was determined using https://sample-size.net, considering an odds ratio (OR) of 2 for one standard deviation of the predictor, and 25% of events (deaths). We performed univariate logistic regressions to assess whether the selected predictors influenced 1-month mortality and to confirm the importance of various clinical scores in predicting this outcome in our cohort. ORs were calculated. Significant predictors were then included in a multiple logistic regression model; then the assumptions were checked. As there was significant multicollinearity between cardiovascular-related predictors, with variance inflation factor (VIF) > 10, only the one that respected the assumption of no multicollinearity was introduced in the final model. In order to investigate whether the model fits were good, McFadden R^2^ was calculated (a value between 0.2 and 0.4 is considered a good model fit). For each multivariate model, we also reported a “whole model” line, which reflects the overall model fit (McFadden R^2^) and global statistical significance, rather than the effect of a single predictor. Internal validation for the final model was performed using the bootstrapping method available in JASP (non-parametric bootstrapping). We generated receiver operating characteristic (ROC) curves for our final model and TRISS score and calculated the area under the curve (AUC), sensitivity, sensibility and Brier score for both of them. Confidence intervals for AUCs were calculated using https://riskcalc.org/ci/ (accessed on 2 December 2025) (Cleveland Clinic).

A *p*-value of <0.05 was considered statistically significant.

## 3. Results

### 3.1. Sample Characteristics

The final cohort comprised 91 patients, with an almost equal gender distribution. Over a quarter of the patients included died within 30 days. The number of fractures varied widely, hence the increased standard deviation; about one sixth of the patients presented with pelvic fractures. We collected data from clinical and blood test records (with a focus on complete blood count, biochemical and coagulation data). Demographic data are summarized in Table 1. We also collected the values of the following scores: RTS, ISS, TRISS, TASH, and ABC transfusion score (Table 2).

We calculated severity scores for our entire cohort. The results are summarized in Table 2.

In order to better characterize the study population, the mechanisms of injury, types of fractures, and distribution of associated lesions were analyzed in detail (Table 3, Table 4 and Table 5).

### 3.2. Simple Logistic Regression

Simple logistic regression was performed to assess different predictors in relation to death by any cause at 1 month after admission. The sample size needed to adequately power a simple logistic regression that expects an OR of 2 for one standard deviation of the predictor and a total of 25% of events (i.e., deaths) was calculated at 90 patients. The analysis results are presented in Table 6. Lower systolic and diastolic blood pressure, reduced SpO_2_ and hemoglobin, together with increased heart rate, glycemia and urea, were significantly associated with mortality based on the value of their respective odds ratio. No significant associations were observed for creatinine, fibrinogen, INR, natremia, kalemia or platelet count.

### 3.3. Multiple Logistic Regression

**Figure A1:**
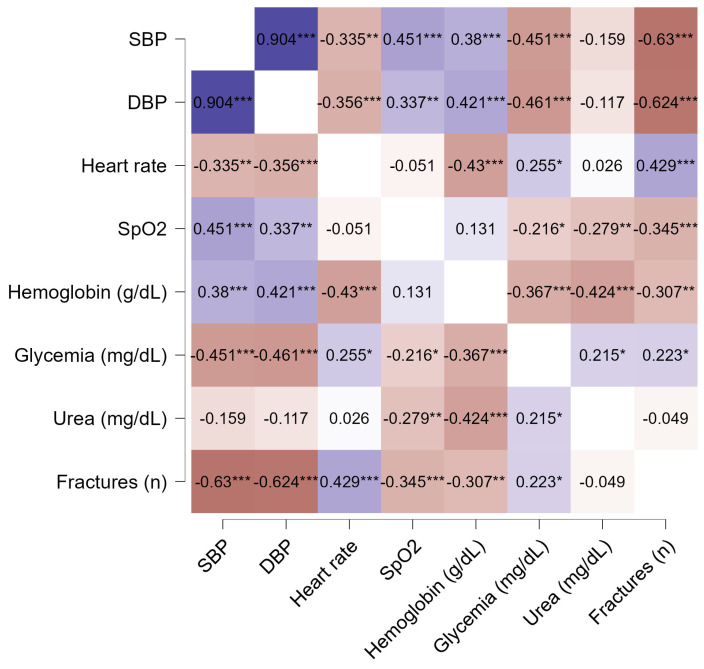
Correlation matrix for the initial multiple logistic regression model (Pearson correlation). * *p* < 0.05, ** *p* < 0.01, *** *p* < 0.001.

Out of all the variables analyzed at Section 3.2, we selected the ones that were statistically significant in predicting death at 1 month after admission and entered them in a multiple logistic regression model in order to investigate which predictors remained significant when adjusting for multiple clinical and paraclinical parameters (Table 7). However, the multicollinearity assumptions were not respected (VIF > 10), with variation inflation factors greater than 10 indicating an interaction between hemoglobin, saturation, and systolic and diastolic arterial pressure, all of these predictors being cardiovascular in nature (Appendix A Table A1, Figure A1).

We first chose SpO_2_, then Hemoglobin, as they were statistically significant in the initial model, but they did not respect the multicollinearity assumption (Appendix A Table A2, Table A3). Moreover, they have several shortcomings, as they could be artificially higher in the case of SpO_2_ after heavy blood loss or lower in the case of hemoglobin if intravenous fluids were administered before hospital admission. We then selected systolic blood pressure (SBP) from the cardiovascular predictors included in the initial model, as it is fairly reliable and easy to measure at admission. SPB also respected the multicollinearity assumption (Appendix A Table A4). The final model therefore included systolic blood pressure, glycemia, serum urea and the number of fractures (Table 8), with only SBP remaining a statistically significant predictor, considering the other confounding cardiovascular predictors were not included. The overall model showed a good fit (McFadden R^2^ of 0.682).

We used bootstrapping (1000 bootstraps) in order to internally validate our model (Table 9). In this case, urea and number of fractures have confidence intervals that cross the threshold of 1, with the other two predictors maintaining statistical significance.

### 3.4. Simple Logistic Regression of Clinical Scores

To assess the predictive value of established trauma scores, we performed simple logistic regression analyses with 30-day mortality as the primary outcome (Table 10). All investigated scores (ISS, RTS, TRISS, ABC score, and TASH score) demonstrated statistically significant associations with mortality. A higher RTS and TRISS were associated with increased survival, while higher total ISS, TASH, and ABC were associated with higher mortality.

The McFadden R^2^ value ranged from 0.275 for the ABC score to 0.560 for the TRISS score, indicating good predictive performance in this cohort. However, all the values were lower than those of the final multiple model (McFadden R^2^ = 0.682). Total ISS showed significance when predicting 30-day mortality, even if we included people with an ISS < 16. When dividing the cohort by this threshold (Appendix A Table A5) we see that in the ISS 8–15 group there were still 2 30-day deaths, confirming that expanding our selection to people with ISS scores lower than 16 included some patients that experienced unfavorable outcomes.

### 3.5. Comparison of Our Model and TRISS

**Figure 2:**
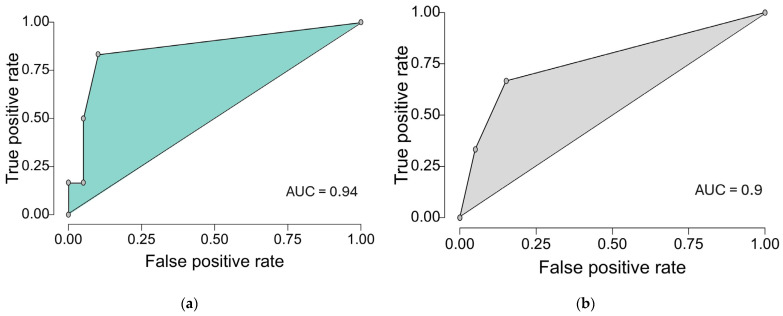
ROC curves of our final multiple logistic regression model that contains the following variables (glycemia, urea, number of fractures, systolic blood pressure), and TRISS when predicting death after 1 month from emergency department admission (cut-off step of 0.2): (**a**) ROC curve of our model predicting 1-month mortality. (**b**) ROC curve of TRISS predicting 1-month mortality.

Finally, we compared our model with the TRISS score. Based on the previous logistic regressions we generated ROC curves both for our final model (Table 8) and TRISS and we calculated the AUC. As we can see in Figure 2, our model yielded similar results to those obtained with the TRISS-based model when considering our cohort, with good model performance (AUC) of 0.94 (CI95% 0.873 to 1), sensitivity of 0.33, specificity of 0.95 and Brier score of 0.077, vs. 0.9 (CI95% 0.815 to 0.985), sensitivity of 0, specificity of 1 and Brier score of 0.086. Considering the small sample size, the TRISS-based logistic model seems to correctly predict all deaths, thus generating these unreliable results for sensitivity and specificity. This does not mean that TRISS failed to predict 30-day mortality in our cohort, but the result is a mathematical error based on predicting all deaths at the cut-off chosen by the statistics software.

## 4. Discussion

In our analysis, we identified a number of clinical and laboratory parameters with prognostic significance for 30-day mortality, as well as the performance of several established scores used in trauma. These observations require interpretation in the context of the existing literature to understand their clinical relevance and limitations.

The first aspect worth discussing concerns the choice of 30-day mortality as the primary outcome, which represented a central element of our analysis. This time point is widely used in the literature to assess post-trauma prognosis, as it provides an overview of trauma severity and clinical course. The use of 30-day mortality as the primary outcome is well supported in the literature in trauma studies. In an analysis conducted by Moore et al., 30-day survival was used as a reference point in the comparative analysis of two groups of patients undergoing massive transfusion, demonstrating the importance of this time point in assessing post-traumatic prognosis [18]. In a randomized trial, Holcomb et al. compared two transfusion strategies using 30-day mortality alongside 24 h mortality, arguing that this approach allows for reporting of both early and late trauma-related deaths [17]. A 2021 Finnish study of a group of patients with severe trauma demonstrated that 30-day mortality is a robust indicator. Using only in-hospital mortality may underestimate the impact of trauma, while extending follow-up beyond 30 days may introduce confounding by including deaths not directly attributable to trauma [21]. Thirty-day mortality is also used in international comparison studies such as that of Ghorbani et al., in which 30-day mortality was defined according to the Utstein template and was used to calculate adjusted survival between two level I trauma centers in Scandinavia [22,23].

Regarding established trauma scores (ISS, RTS, TRISS), our analysis confirms several important observations. They remain essential tools in assessing patient prognosis. In our cohort, the ISS value was directly correlated with 30-day mortality, confirming previous findings in the literature, where this score, used as an anatomical marker of injury severity, has been shown to be associated with the risk of death [24,25,26]. The median ISS was 17 (IQR 13–35), compared with the value of 26.5 (IQR 17–41) reported by Holcomb et al. in 2015 in the PROPPR study [17], which is most likely due to the distinct inclusion criteria and regional case-mix particularities. We believe that this aspect does not diminish the relevance of the results since the objective of the analysis was represented by 30-day mortality, a valid benchmark regardless of the ISS threshold used. In contrast, the other two scores (RTS and TRISS) showed an inverse association with mortality, which is explained by their method of construction: a higher value indicates a better physiological status, with preserved vital functions [9,10,11,12]. Within the studied cohort, the mean RTS of 7 ± 1.653 is comparable to other cohorts in the international literature (7.30 ± 1.15 in a study conducted by Mohammed et al. in 2016 [27]), which suggests that the population analyzed by us has a similar initial physiological profile and supports the validity of the interpretation of the results. Regarding TRISS, there is a particular aspect that is worth mentioning and analyzing: developed in 1987, quickly becoming the gold standard for assessing the quality of care in trauma centers and for comparisons between populations, it is based on a population and medical practice different from those of today [12]. Subsequent studies have highlighted that the original formula may overestimate or underestimate the probability of survival in other territories or in the context of modern scientific advances. Thus, in 2010, in an analysis that included over 400,000 patients from three large international trauma registries, Philip Schluter confirmed the importance of periodically updating this score, demonstrating that the revised formulas provide superior performance to the original TRISS score [28]. Consistent with these observations, in our cohort the overall performance of TRISS was only moderate, suggesting that its accuracy may be limited when applied outside the geographical and scientific context in which it was developed.

Another aspect worth highlighting is the etiological profile of trauma in Romania, which differs markedly from that reported in the populations on which the established scores were originally developed. While in the United States and other Western regions a considerable proportion of severe trauma cases result from firearms or other penetrating mechanisms, data from European trauma registries indicate a predominance of blunt injuries, particularly those related to road traffic accidents. This distinct pattern, also reflected in the typology of our cohort, underscores the need for local validation of prognostic models and their adaptation to the epidemiological and infrastructural characteristics of the Romanian trauma care system [3,29].

At the same time, in our cohort, a significant association with mortality was also observed for the ABC and TASH scores, suggesting that they may have an indirect prognostic value for mortality. Although these scores were developed to anticipate the need for massive transfusion in polytrauma patients, this statistical association may be explained by the fact that both are based on clinical and paraclinical parameters that reflect the severity of hemorrhagic shock, a key determinant of vital prognosis. However, this finding should be regarded as a secondary observation, since mortality prediction is not the primary objective of these two scores, which are only surrogate markers of vital risk. In the multicenter validation study of the ABC score conducted by Cotton et al. in 2010, the authors highlighted the high risk of death among patients requiring massive transfusion [16], which makes the score function indirectly as a prognostic marker. Similarly, Yucel’s study, which laid the basis for the development of the TASH score, emphasized that it can also be considered a surrogate marker for life-threatening bleeding [19]. Maegele et al. also noted in their revalidation study of the TASH score that uncontrolled bleeding remains a major cause of death in severe trauma [20].

Despite the fact that established trauma scores demonstrated a statistically significant association with mortality, the overall recorded performance was only moderate, with McFadden R^2^ values between 0.275 and 0.560. Extending the comparison, we also analyzed our final multivariable model, which included SBP, blood glucose, urea and the number of fractures, yielding a McFadden R^2^ of 0.682. These results should be interpreted with caution, as our model is based on an exploratory study developed on a relatively small sample, with a high risk of overfitting. Nevertheless, the exclusive use of simple, readily available parameters exhibits very good potential for future validation on larger, multicenter cohorts. Although SBP remained the only statistically significant cardiovascular predictor in the model, the overall performance of the model reached that of established scores. This result does not imply that the other parameters lack clinical relevance, but rather that their independent effects did not reach statistical significance when considered alongside systolic blood pressure—a limitation likely due to the small cohort size and the interaction between the variables. Systolic blood pressure is considered a direct marker of hemodynamic instability and hemorrhagic shock, as it reflects, on the one hand, circulating blood volume depletion and, on the other hand, cardiovascular compensatory capacity. This may explain its superior prognostic performance compared with secondary biological or metabolic markers. The prognostic importance of the SBP has been studied several times over the years. A study published in 2012 in *Resuscitation* journal, which analyzed 3444 patients with penetrating trauma, showed that mortality in patients presenting with SBP between 90 and 109 mmHg and with an SBP between 90 and 89 mmHg is two and four times higher, respectively, compared with the reference group of patients with an SBP between 110 and 129 mmHg [30]. Moreover, this study recommended directing patients with penetrating trauma and SBP < 110 mmHg to a high-level care trauma center [30]. Similarly, a recent analysis of 8798 patients confirmed that dynamic monitoring of SBP provides additional prognostic value [31]. One particular aspect worth mentioning is that, in our model, blood glucose showed a positive association with mortality the estimated effect remained consistent with that previously reported in literature [32,33,34], suggesting that it may be further confirmed in larger cohorts. Pathophysiologically, hyperglycemia at presentation is frequently associated with activation of the post-traumatic neuroendocrine stress response and may reflect the severity of shock and the degree of tissue hypoperfusion. Similarly, the association of urea with mortality in the univariate analysis may reflect early renal hypoperfusion and a hypovolemic state during initial shock phases, as well as the intensification of protein catabolism induced by post-traumatic metabolic stress. In a study conducted in Switzerland involving 555 patients, blood glucose on hospital admission proved to be an independent predictor of in-hospital mortality [33]. Furthermore, in 2019, Winkelmann et al. reported that blood glucose level on hospital admission was an independent predictor of shock and mortality in polytrauma patients [34].

Nonetheless, there is a relevant methodological aspect to be addressed concerning the choice of ISS ≥ 8 as the threshold for patient inclusion in the cohort, despite international literature defining polytrauma as ISS ≥ 16. We selected this threshold to avoid excluding cases which, despite presenting a moderate ISS at initial evaluation, might subsequently experience an unfavorable course. We believe that this approach may reflect actual clinical practice in a more accurate manner, where prognosis depends on multiple factors beyond the anatomical injury score. Nevertheless, this choice represents a limitation of our study and underscores the need for and importance of validation in larger cohorts.

The present study has several limitations that should be acknowledged. First, the relatively small sample size may explain why some variables only showed trends toward association without reaching conventional statistical significance, as well as the limited statistical power of the analyses performed. Furthermore, the retrospective design, relying on pre-existing data from clinical evaluation sheets and hospital electronic information system, introduces both a selection bias—by including only those patients with complete, available data—and a limitation caused by the lack of uniformity in data collection. In addition, by being a single-center study, the results cannot be generalized without caution to other populations or clinical contexts.

The single-center design of the study limits the generalizability of the results, as the organization of trauma care, pre-hospital response times, local protocols, and resource availability may influence patient outcomes independently of the analyzed scores. The specific characteristics of the regional trauma network may thus modify the performance of the model in other clinical settings, highlighting the need for validation in multicenter cohorts.

There is a significant risk of optimism bias, as the model was built from our cohort, even though it passed internal validation after resampling through bootstrapping, with the same predictors being statistically significant in the bootstrapped model as in the final model. Considering that the final model has 4 predictors and the number of deaths is 24, the model is likely overfitted and should be validated on a sample with at least 40 events. While sufficient for univariate analysis, a sample of 91 patients is not adequately powerful for a multivariate logistic model.

Finally, the proposed model has not been externally validated, which means the results should be considered specific to this geographical area and case mix. Future research should especially focus on finding the influence of non-cardiovascular predictors. Metabolic data need better validation in the context of trauma patients and the acute changes caused by blood loss. Glycemia is an interesting predictor that should be monitored for inclusion in future multicentric models, as hyperglycemia is associated with poorer outcomes for these patients in both our model and in literature. We believe that these limitations open perspectives for future research, highlighting the need to extend the analysis to larger, multicenter and prospective cohorts that would allow for both validation and recalibration of the proposed model to enhance its clinical applicability. After these steps are taken, the new logistic regression coefficients obtained after application and validation on larger datasets could be introduced into an equation. A helpful triage threshold could then be generated. In order to improve predictive accuracy and to develop a prognostic score tailored to the local population, the integration of additional variables may prove highly valuable.

Beyond its exploratory value, our model has the potential to be practically applied in the initial assessment of polytraumatized patients in Romanian emergency departments. The choice of simple clinical and paraclinical variables, available in the first minutes of admission—systolic blood pressure, blood glucose, urea and number of fractures—offers the possibility of rapid use of the model without the need for complex investigations or advanced technological resources. In a medical system where trauma centers are numerically limited and the load of emergency departments is high, such an algorithm could support the triage decision, identifying early patients with increased vital risk and directing them to regional centers capable of ensuring complete multidisciplinary management.

Moreover, the application of a prediction model adapted to Romanian realities can contribute to the standardization of the initial assessment process and to increasing the uniformity of medical care in major trauma. Its implementation in emergency department workflows would allow for a more efficient use of resources (transfusions, interhospital transport, intensive care), reducing the gaps between different levels of care. From this perspective, prospective validation and multicenter expansion of the model would represent a natural step towards the development of a local prognostic score, adapted to the national epidemiological and infrastructural context, with the potential to become a practical decision-support tool in the Romanian trauma network.

## 5. Conclusions

In this study on patients presenting to the Emergency Department as trauma victims, SBP was identified as an independent predictor of 30-day mortality, with the overall multivariable model demonstrating excellent discriminative ability. The results emphasize that simple, commonly available clinical parameters could contribute to risk stratification, highlighting the prognostic importance of hemodynamic instability and metabolic response. However, this study, conducted on a relatively small cohort, is exploratory in nature, which makes validation of the results imperative through a larger, multicenter study with a confirmatory design in order to accurately confirm the observed relationships and enable the development of a new score.

## Figures and Tables

**Table 1:** Baseline characteristics of the study cohort.

Characteristic		n = 91
Sex		
Male		46 (50.55%)
Female		45 (49.45%)
30-day mortality—n (%)		
Yes		24 (26.37%)
No		67 (73.63%)
Pelvic fractures—n (%)		
Yes		14 (15.38%)
No		77 (84.62%)
Femoral fracture—n (%)		
Yes		8 (8.8%)
No		83 (91.20%)
	Mean	Standard Deviation
Age	54.2	20.6
Systolic Blood Pressure (mmHg)	111.92	32.18
Diastolic Blood Pressure (mmHg)	63.07	18.19
Heart Rate (beats per min)	100.58	21.56
SpO_2_ (%)	94.87	5.74
Hemoglobin (g/dL)	12.00	2.56
Thrombocytes (×10^3^/µL)	228.89	74.3
Glycemia (mg/dL)	144.30	63.93
Na^+^ (mEq/L)	140.06	3.23
K^+^ (mEq/L)	4.06	0.61
Urea (mg/dL)	43.41	26.66
Serum creatinine (mg/dL)	1.20	1.74
Fibrinogen (mg/dL)	339	135.49
INR	1.37	1.685
aPTT (s)	30.81	41.1
Fractures (n)	5	5.777

**Table 2:** Trauma severity scores of the study cohort.

Characteristic	Mean	Standard Deviation	Median (IQR)
Total ISS	24.011	15.262	17 (22)
RTS	7	1.653	8 (2)
TRISS	75.698	34.585	96.3 (56.8)
TASH score	6.363	6.492	3 (9)
	**Value**	**Number (percent)**	
ABC score	0	61 (67%)	
	1	6 (6.6%)	
	2	14 (15.4%)	
	3	10 (11%)	

**Table 3:** Mechanism of injury and fracture distribution.

Characteristic	n = 91 (%)
**Mechanism of Injury**	
Blunt trauma	87 (95.6%)
Penetrating trauma	4 (4.4%)
**Type of accident**	
Road traffic accident	65 (71.4%)
Fall from same level	8 (8.8%)
Fall from height	6 (6.6%)
Compression/crush trauma	6 (6.6%)
Other	2 (2.2%)

**Table 4:** Distribution of injured anatomical regions in the study cohort.

Injured Anatomical Region	n = 91 (%)
Head and neck	14 (15.4%)
Face	6 (6.6%)
Thoracic region	42 (46.2%)
Abdominal/pelvic contents	23 (25.3%)
Extremities/pelvic girdle	49 (53.8%)
External (soft-tissue injuries)	10 (11%)

Percentages refer to the total cohort (n = 91); categories are not mutually exclusive, as multiple regions could be affected in the same patient.

**Table 5:** Distribution of the number of fractures per patient in the study cohort.

Number of Fractures per Patient	n = 91 (%)
<3 fractures	47 (51.6%)
3–5 fractures	18 (19.8%)
6–10 fractures	14 (15.4%)
>10 fractures	12 (13.2%)

Fracture categories represent the total number of radiologically confirmed fractures per patient at presentation. Percentages are calculated based on the total cohort (n = 91).

**Table 6:** Simple Logistic Regressions predicting 30-Day Mortality.

Characteristic	Odds Ratio	*p*-Value
**Systolic Blood Pressure (mmHg)**	**0.919 (0.887 to 0.953)**	**<0.001**
**Diastolic Blood Pressure (mmHg)**	**0.866 (0.815 to 0.919)**	**<0.001**
**Heart Rate (bpm)**	**1.028 (1.005 to 1.051)**	**0.015**
**SpO_2_ (%)**	**0.887 (0.815 to 0.966)**	**0.003**
**Hemoglobin (g/dL)**	**0.510 (0.378 to 0.688)**	**<0.001**
Thrombocytes (m/mm^3^)	0.997 (0.991 to 1.004)	0.44
**Glycemia (mg/dL)**	**1.027 (1.013 to 1.042)**	**<0.001**
Na^+^ (mEq/L)	1.037 (0.897 to 1.199)	0.62
K^+^ (mEq/L)	0.640 (0.299 to 1.373)	0.25
**Urea (mg/dL)**	**1.022 (1.004 to 1.040)**	**0.016**
Serum creatinine (mg/dL)	0.973 (0.727 to 1.302)	0.85
Fibrinogen	0.996 (0.990 to 1.001)	0.1
INR	0.904 (0.608 to 1.346)	0.62
aPTT	1.015 (0.987 to 1.044)	0.29

Values are presented as odd ratio (OR) with 95% confidence intervals per unit increase in each predictor. All parameters represent admission values at Emergency Department presentation.

**Table 7:** Multiple logistic regression model including all significant predictors from univariate analysis.

Variable	Odds Ratio	*p*-Value	McFadden R^2^
Whole model		<0.001	0.786
SpO_2_ (%)	1.167 (1.003 to 1.357)	0.046	
Hemoglobin (g/dL)	0.304 (0.111 to 0.831)	0.02	
Glycemia (mg/dL)	1.042 (1.003 to 1.083)	0.035	
Urea (mg/dL)	1.026 (0.965 to 1.091)	0.41	
Fractures (n)	1.167 (0.960 to 1.418)	0.12	
Diastolic Blood Pressure (mmHg)	1.079 (0.919 to 1.266)	0.35	
Heart Rate (bpm)	0.937 (0.872 to 1.006)	0.07	
Systolic Blood Pressure (mmHg)	0.914 (0.826 to 1.012)	0.08	

**Table 8:** Final multiple logistic regression model after adjustment for multicollinearity.

Variable	Odds Ratio	*p*-Value	McFadden R^2^
Whole model		<0.001	0.682
Glycemia (mg/dL)	1.021 (1.006 to 1.036)	0.007	
Urea (mg/dL)	1.015 (0.978 to 1.055)	0.43	
Fractures (n)	1.106 (0.990 to 1.235)	0.07	
Systolic Blood Pressure (mmHg)	0.944 (0.920 to 0.969)	<0.001	

**Table 9:** Bootstrapped OR and confidence intervals (1000 bootstraps) for the final logistic regression model.

Variable	Odds Ratio
Glycemia (mg/dL)	1.022 (1.009 to 1.049)
Urea (mg/dL)	1.015 (0.983 to 1.122)
Fractures (n)	1.119 (0.958 to 1.422)
Systolic Blood Pressure (mmHg)	0.942 (0.884 to 0.961)

**Table 10:** Univariate logistic regression of trauma severity (ISS, RTS, TRISS) and transfusion-related scores (ABC, TASH) for 30-day mortality.

Characteristic	Odds Ratio	*p*-Value	McFadden R^2^
TOTAL ISS	1.113 (1.065 to 1.162)	<0.001	0.339
RTS	0.286 (0.177 to 0.462)	<0.001	0.469
TRISS	0.936 (0.913 to 0.959)	<0.001	0.560
TASH score	1.255 (1.142 to 1.379)	<0.001	0.301
ABC score	3.385 (2.041 to 5.616)	<0.001	0.275

## Data Availability

This article does not include any additional primary data besides the information already presented in the case report section.

## Appendix A

**Table A1:** Multicollinearity diagnostics for the initial multiple logistic regression model.

	Tolerance	VIF
**SpO_2_**	0.005	216.195
**Hemoglobin (g/dL)**	0.009	111.279
**Glycemia (mg/dL)**	0.025	40.260
**Urea (mg/dL)**	0.120	8.333
**Fractures (n)**	0.264	3.786
**DBP**	0.012	80.297
**Heart rate**	0.014	70.481
**SBP**	0.010	96.254

**Table A2:** Multicollinearity diagnostics for a final model including hemoglobin as a cardiovascular predictor.

	Tolerance	VIF
**Hemoglobin (g/dL)**	0.030	33.114
**Glycemia (mg/dL)**	0.052	19.192
**Urea (mg/dL)**	0.223	4.478
**Fractures (n)**	0.342	2.921

**Table A3:** Multicollinearity diagnostics for a final model including SpO_2_ as a cardiovascular predictor.

	Tolerance	VIF
**SpO_2_**	0.051	19.647
**Glycemia (mg/dL)**	0.101	9.887
**Urea (mg/dL)**	0.191	5.231
**Fractures (n)**	0.387	2.587

**Table A4:** Multicollinearity diagnostics for a final model including SBP as a cardiovascular predictor.

	Tolerance	VIF
**Glycemia (mg/dL)**	0.103	9.663
**Urea (mg/dL)**	0.224	4.471
**Fractures (n)**	0.433	2.312
**SBP**	0.110	9.059

**Table A5:** Contingency table of 30-day mortality divided by ISS score.

	30-Day Mortality		
ISS Score	0	1	Total
**ISS 16 and over**	**43**	**22**	**65**
**ISS under 16**	**24**	**2**	**26**
**Total**	**67**	**24**	**91**
